# International Committee for Post-Graduate Medical Education

**Published:** 1911-01-21

**Authors:** 


### INTERNATIONAL COMMITTEE FOR POST-GRADUATE MEDICAL EDUCATION.

The first general meeting of the International Committee for Post-Graduate Medical Education was held in the Kaiserin Friedrich Haus, at Berlin, in October last, Prof. Dr. Waldeyer in the chair.

The following delegates attended: Sir Donald MacAlister, President, General Medical Council (London), Dr. Blondel (Paris), Fleet-Surgeon J. H. A. Clayton (London), Dr. Paul Courmont (Lyon), Joseph C. Grew, Secretary of the American Embassy (Berlin), Hofrat Prof. Dr. E. von Grosz (Budapest), Dr. J. Hiteff (Sofia), Prof. Dr. Johannessen (Christiania), Prof. Dr. W. W. Keen (Philadelphia), Prof. Dr. R. Kutner (Berlin), Dr. P. von der Mühll (Basle), A. Nörgaard, Secretary of the Royal Danish Legation (Berlin), Government Privy Counsellor Tilmann (Berlin), Ministerial Counsellor Dr. Ludwig von Toth (Budapest), Privy Medical Counsellor Prof. Dr. Waldeyer (Berlin), Medical Counsellor Dr. Wawrinsky (Stockholm), Prof. Dr. K. F. Wenckebach (Groningen).

The proceedings were transacted in French.

The General Secretary (Professor Kutner) reported the following changes in the composition of the committee :—

America.—The following were appointed delegates for America: Dr. G. N. Miller (New York), Dr. Ed. Quintard (New York), Dr. W. S. Thaeyer (Baltimore), Brigadier-General G. H. Torney, Surgeon-General of the United States Army (Washington), Dr. C. F. Stokes, Surgeon-General of the United States Navy (Washington).

Belgium.—Prof. Dr. Firket (Liége) and Dr. Dejace (Liége) have retired from the International Committee, not having received the necessary assistance from the Government in their efforts in behalf of post-graduate medical education.

England.—Dr. F. W. Pavy (London) has retired from the International Committee on account of his advanced age. Dr. F. Pickthorn (London) has also retired, because of his transfer to active service in the Navy.

France.—To the previous French delegates have been added Prof. Dr. Roux (Paris), Directeur de l'Institut Pasteur, and Dr. Blondel, Directeur du Bureau des renseignements de l'Université de Paris. Furthermore, the French delegation, in agreement with the previous deputy, Prof. Tripier (Lyons), requested to be permitted to nominate as delegate in his stead Prof. Courmont (Lyons), to which the meeting gave its consent.

Switzerland.—Dr. O. von der Mühll (Basle) has been nominated by the Swiss Medical Association as its representative on the International Committee.

Furthermore, the Secretary-General stated that, according to a communication from Geheimrat von Rein, the Imperial Medical Council in Russia has appointed a commission to organise post-graduate medical education in that country.

A central organisation is also in course of preparation in America, thanks to the efforts of Messrs. Miller and Kast, but it is not yet finally concluded. In this connection the Secretary-General expressed the urgent wish that in other countries also central organisations would soon be created for post-graduate medical education, such as up to the present only exist in Germany and Hungary.

In the discussion Dr. Blondel made the proposal, in the event of a delegate being prevented from attending a meeting, that he should be allowed to send a deputy, who should have the same rights as the official delegate. Mr. Blondel's proposal proved to be unnecessary, for the reason that provision has already been made for substitution in Par. 11, subsection 1 of the statutes. Finally, the Secretary-General read a letter from the American Delegation, in which the wish was expressed that the title of the International Committee in English translation should in future be "The International Committee for Post-Graduate Medical Education." In the same communication it was requested that Prof. Dr. Kast, whom the American Government had not nominated, because he had not at the time been naturalised, should be elected as an adjunct of the International Committee.

In connection with the latter wish, the Secretary-General alluded to the services of Prof. Kast in the cause of post-graduate medical instruction in America and recommended the election. It was true that the number of five American delegates had been already attained; so that Dr. Kast, inasmuch as the adjunct must really always be a member, could only be elected as an exception and without the right to vote. The International Committee resolved accordingly, and nominated Dr. Kast as a member of the International Committee and as its American adjunct, but without the right to vote. The application regarding the English translation of the name, "The International Committee for Post-Graduate Medical Education," was also accepted.

### The Secretary's Report.

The Secretary-General presented a report upon the proposed collective investigation, and stated that hitherto no objections to the form of the list of questions had been raised by the delegates of the various countries. An exhaustive discussion took place regarding the list of questions and the manner of carrying on the collective investigation. It was finally resolved that the Secretary of the committee be authorised to prepare the list of questions in the German, English, and French languages, and to send it to the delegates of the International Committee in the various countries. The completed manuscript must be approved by the delegation of the respective country, and must then be forwarded to the Secretary-General of the International Committee, who will then sort out the whole of the material and compile it in one collective work, which would be most appropriate if published in the German, English, and French languages.

The Secretary presented a request to the members of the International Committee to send in reports to the Information Department, in the Kaiserin Friedrich Haus, at perhaps quarterly or semi-annual intervals, regarding the courses and lectures for the purpose of post-graduate medical education in the various countries. The meeting expressed its willingness to comply with the request of the Secretary-General. The object is to create an office from which the medical men of all countries can obtain free information regarding post-graduate education in the various civilised lands at any time. The discussion of the details of this organisation, which was agreed upon by the meeting as being entirely expedient, was reserved for the next meeting.

For the purpose of covering the material expenses of the business office (printed matter, postage, etc.), according to the opinion of the management, a member's fee will have to be paid to the International Committee. The Secretary-General pointed out that in the case of the International Union against Tuberculosis the sum of 100 marks was paid annually by each country for each delegate. After an exhaustive discussion it was resolved that the delegates of the individual countries should propose to their Governments the payment of an annual member's fee of 50 marks for each delegate. April 1, 1911, was fixed as the date for the transmission of a reply to the Bureau.

The Hungarian delegates moved : "To what extent can the training and post-graduate education of the medical men in the various civilised countries in respect to the permission to carry on practice be considered as of equal value ? "

Professor von Grosz stated the reasons for the application, and proposed to nominate a Commission, which should occupy itself in ascertaining the prescribed conditions for carrying on practice and with the question of the mutual recognition of foreign diplomas. Inasmuch as the majority of the meeting was of opinion that the application had already been settled Prof. von Grosz withdrew his motion. It was finally decided, without a definite resolution being passed, to hold the next meeting of the International Committee in conjunction with the approaching International Medical Congress in London, in which connection the President expressed the wish that by that time the work of the collective investigation decided upon could be settled.

We herewith publish the list of questions with reference to lectures. Any information on the points alluded to will be welcomed, and we shall be glad if secretaries for graduate classes in this country will kindly forward particulars of such classes as soon as possible.

List of questions for courses and lectures during the period from January 1, 1911, to July 1, 1911.—There will take place during the period from January 1, 1911, to July 1, 1911, courses and lectures for the after-college training of physicians : 1. Where? 2. When? 3. Can foreigners participate? 4. What subjects are taken into consideration in the courses and lectures? 5. Are the courses and lectures paid for, or gratis? 6. If paid for, how much do the fees amount for the single course? 7. To whom are interested parties to be referred for further particulars (place of business)?

